# Antimicrobial stewardship in South Africa: a scoping review of the published literature

**DOI:** 10.1093/jacamr/dlz060

**Published:** 2019-11-28

**Authors:** Sarentha Chetty, Millidhashni Reddy, Yogandree Ramsamy, Anushka Naidoo, Sabiha Essack

**Affiliations:** 1 Discipline of Pharmaceutical Sciences, School of Health Sciences, Westville Campus, University of KwaZulu-Natal, Durban, South Africa; 2 Essential Medicine Consulting, Durban, South Africa; 3 Department of Medical Microbiology, Prince Mshiyeni Memorial Hospital - National Health Laboratory Services, Antimicrobial Research Unit, University of Kwazulu-Natal, Durban, South Africa; 4 Centre for the AIDS Programme of Research in South Africa (CAPRISA), University of KwaZulu-Natal, Durban, South Africa; 5 Antimicrobial Research Unit, College of Health Sciences, University of KwaZulu-Natal, Durban, South Africa

## Abstract

**Objectives:**

To map published data of antimicrobial stewardship (AMS) interventions that are currently being carried out in hospitals and clinics in the public and private health sectors of South Africa in line with the antimicrobial resistance (AMR) strategy of South Africa.

**Methods:**

A systematic scoping review was conducted to identify AMS initiatives in the public and private health sectors of South Africa for the period 1 January 2000 to 31 March 2019. An electronic search of databases was made including PubMed, Scopus, a key medical journal (*South African Medical Journal*), University of KwaZulu-Natal (UKZN) WorldCat iCatalogue and AMR networks: Federation of Infectious Diseases Societies in South Africa (FIDSSA). Reference lists of published articles were also reviewed for inclusion. Keywords included ‘antimicrobial antibiotic stewardship South Africa’.

**Findings:**

Of a total of 411 articles, using a stepwise screening process, 18 articles were selected for inclusion in the review. The interventions/initiatives were divided into four broad categories: (i) AMS intervention: prescription audits and usage; (ii) AMS intervention: education and its impact; (iii) other AMS interventions; and (iv) the role of different healthcare professionals in AMS.

**Conclusions:**

The data identifies a need for and the value of AMS in both the public and private health sectors of South Africa. Initiatives are carried out across both sectors but more attention needs to be focused on AMS implementation in line with the National AMR Strategy of South Africa. Collaboration between the different sectors will aid in overcoming the AMR challenge.

## Introduction 

The introduction of antibiotics in the 1940s revolutionized modern medicine. However, due to the appearance of resistance to all known classes of antibiotics, the human race faces a future with untreatable infectious diseases.^1^ In addition to the threat to the human population, antimicrobial resistance (AMR) adversely influences a country’s economy owing to increasing healthcare costs involved in treating infections caused by MDR organisms.^1^^,^^2^ The development of AMR is directly attributable to the inappropriate use of and exposure to antimicrobial agents. Upon exposure to antimicrobial agents, bacteria are continuously under pressure to evolve to ensure survival of the species.^1^ Evidence suggests that there has been an overall decrease in the effectiveness of all antibiotics globally.^1^ Coupled with the appearance of AMR, the world faces a dwindling pool of antimicrobial agents, with very few new antibiotics under development. With very limited antimicrobial agents on the horizon, preservation of existing antibiotics is critical.^3^ AMR has justly been proclaimed a global health emergency. To avoid acceleration towards a ‘post-antibiotic era’, the urgency of intervention cannot be overstated. In September 2016, a political declaration was sanctioned by the Heads of State of the United Nations General Assembly declaring its pledge to address the global problem of AMR.^4^

In South Africa (SA), the national department of health (NDOH) has recognized the threat of AMR, hence the drafting of a National AMR Strategy Framework, with the view of managing and curtailing AMR.^5^ The National AMR Strategy Framework is a comprehensive approach with accountability, roles and responsibilities that is aimed at tackling AMR.^5^ Through prudent and appropriate antimicrobial use, the efficacy of such agents can be preserved for future human and animal use. Surveillance, infection prevention and control (IPC) and antimicrobial stewardship (AMS) are three pillars critical in preventing AMR.^6–8^

The cornerstone of improved antimicrobial usage is a robust AMS programme (ASP). AMS ‘refers to coordinated interventions designed to improve and measure the appropriate use of antimicrobial agents by promoting the selection of the optimal antimicrobial drug regimen including dosing, duration of therapy and route of administration’.^9^^,^^10^ Evidence has demonstrated that AMS inventions have reduced duration of therapy and hospital stay without adverse patient outcomes, which would potentially result in a reduction in colonization and subsequent infection of patients with MDR bacteria.^11^^,^^12^

The South African healthcare system consists of a public/state sector and a private healthcare sector. The public sector is largely funded by government,^13^^,^^14^ whereas the private sector is predominantly funded through individuals and medical insurance schemes. The majority of South Africans (82.5%–84%) access healthcare via the public sector.^14^^,^^15^ The NDOH is responsible for drafting and enforcing national health policy. There are nine provincial departments of health, each responsible for carrying out national policy and public health service delivery within the province. The public sector comprises a three-tiered hospital system: tertiary, regional and district, as well as a primary healthcare (PHC) level, and community healthcare centres (CHCs). Healthcare delivery at the PHC and CHC level is predominantly provided by nurses at a clinic.^13^

The private sector is made up of GPs, specialists and private hospitals.^13^ Three hospital groups, Netcare, Mediclinic and Life, dominate and account for approximately 80% of the private sector.^16^ At present, commendable efforts are being made in both sectors towards AMS interventions.

The primary aim of this scoping review is to map the existing literature on the AMS interventions currently being carried out in hospitals and clinics in the respective sectors. Community-based services were excluded. The objectives were to: (i) describe AMS initiatives in SA; and (ii) evaluate and compare AMS interventions in the public and private sectors with a view to identifying adaptable good practice.

## Methods

### Ethics

Ethical approval was not required as this was a review of the published literature.

### Scoping review framework

The authors adopted the scoping review framework of Arksey and O’Malley^17^ and Levac *et al.*^18^ The framework consists of: (i) identifying the research question; (ii) identifying relevant studies; (iii) study selection; (iv) charting the data; and (v) collating, summarizing and reporting the results.

#### Identifying the research question

The research question was: ‘What is known from existing literature about the AMS interventions carried out in the public and private sectors in SA?’ The research subquestions consisted of the following:

- What are the AMS initiatives currently carried out in hospitals and clinics in the public and private healthcare sectors in SA?
- Is there a difference in the AMS interventions carried out in the public and private healthcare sectors in SA?

##### Eligibility of the research question

An adapted population, intervention, comparison, outcomes and study setting (PICOS) framework was used to determine the eligibility of the research question (Table 1).

**Table 1.** PICOS framework for determination of the eligibility of the research question

Criteria	Determinants
Population	patients in hospitals/clinics from the public and private sectors
Intervention	evidence of AMS interventions in hospitals/clinics in the public and private sectors
Comparison	previous AMS activity
Outcomes	benefits, advantages and disadvantages of AMS interventions in improving quality of patient care and reducing AMR
Study setting	SA

#### Identifying relevant studies

A systematic scoping review was conducted to identify AMS initiatives in the public and private health sectors of SA for the period 1 January 2000 to 31 March 2019. Peer-reviewed research articles, review articles and grey literature pertaining to the research question were included. Electronic databases used to source the data were PubMed, Scopus, a key medical journal: *South African Medical Journal* (*SAMJ*), University of KwaZulu-Natal (UKZN) WordCat iCatalogue, reference lists, networks: Federation of Infectious Diseases Societies in South Africa (FIDSSA), conferences and grey literature. Reference lists of articles selected were also reviewed for inclusion.

#### Study selection

A comprehensive systematic title screening of the databases using the keywords ‘antimicrobial antibiotic stewardship South Africa’ was conducted by one reviewer (S.C.). The search results were exported to an EndNote X8.2 database and duplicates were removed. The EndNote library was then shared with the three other reviewers (A.N., M.R. and Y.R.). All titles and abstracts were screened by at least two reviewers (S.C. and A.N., S.C. and M.R. or S.C. and Y.R.) using predetermined inclusion/exclusion criteria (Table 2), with a third reviewer (S.E.) resolving any disagreements.

**Table 2.** Inclusion and exclusion criteria

Category
Inclusion criteria
antibiotic stewardship interventions in SA
antibiotic stewardship interventions in a hospital and/or clinic
antimicrobial/antibiotic stewardship in the human population/humans
language restricted to English
studies published from 1 January 2000 to 31 March 2019
Exclusion criteria
AMS interventions outside of SA
AMS in animals and agriculture
antimicrobial surveillance
IPC, reviews, commentaries or expert opinion on AMS without active AMS interventions
AMS in the community sector
studies published prior to January 2000
studies published after 31 March 2019

##### Eligibility criteria

The inclusion/exclusion criteria (Table 2) were developed based on the research question to enable correct selection of the relevant articles.

**Figure 1.**
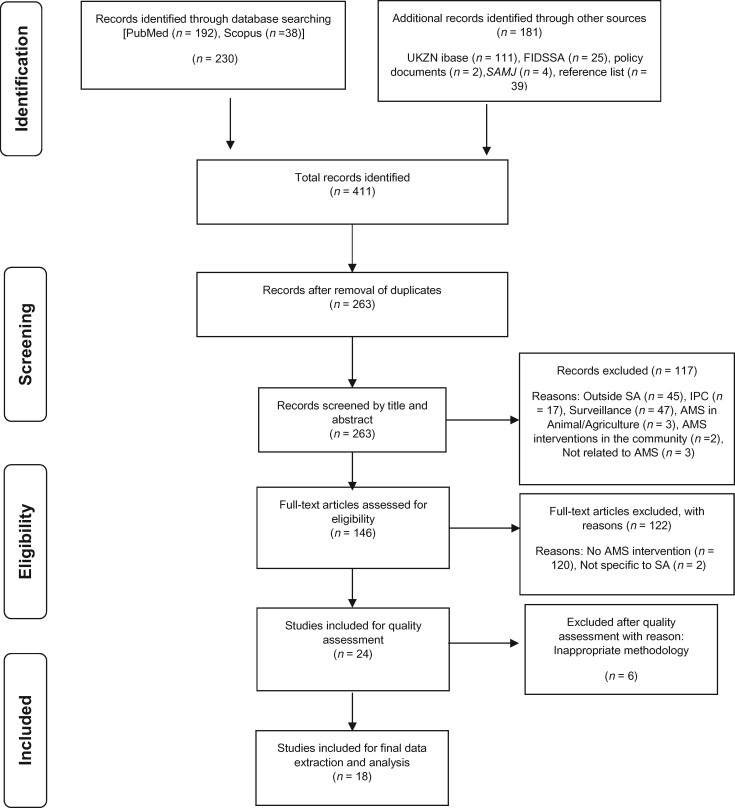
PRISMA 2009 flow diagram representing records identified. Adapted from Moher *et al.*^88^

The full-text articles of the included studies underwent a further stepwise screening process for eligibility. This was conducted by at least two reviewers (S.C. and A.N., S.C. and M.R. or S.C. and Y.R.). These studies were then analysed according to eligibility criteria and quality assessment. The Mixed Methods Appraisal Tool (MMAT), version 2011 was used to assess the quality of the studies.^19^^,^^20^ The selection procedure is detailed and summarized in a modified PRISMA chart (Figure 1).

#### Charting the data (data extraction and analysis)

The adapted PRISMA 2009 tool was used to map the relevant articles retrieved. A standardized data extraction sheet based on an adapted PICOS framework (Table 1) was used to guide eligibility of the studies selected (Table 3). The table included the following fields: author, year, study location, study design, study population, sector (public/private), aims of the study, intervention and summary of results. MMAT, version 2009^19^^,^^20^ was used to assess the quality of the included studies.

**Table 3.** Summary of studies identified

Author, year	Study location	Study design	Study population (sector)	Aims of the study	Intervention	Summary of results
Boyles *et al.*, 2013^35^	Two general medical wards in an academic teaching hospital, Groote Schuur Hospital in the Western Cape	Observational	General medical patients who had been prescribed antibiotics (public)	Introduction of a two-component intervention aimed at reducing antibiotic consumption for better patient outcomes	Antibiotic prescription chart and a weekly antibiotic stewardship ward round	592.0 DDDs were prescribed per 1000 inpatient days (control period) versus 475.8 DDDs per 1000 inpatient days (intervention period) There was a 19.6% decrease in antibiotic volume following the intervention There was a 35% cost reduction in the pharmacy’s antibiotic budget An increase in laboratory tests was noted due to requests for procalcitonin levels There was no difference in inpatient mortality or 1 month readmission rates following the intervention
Boyles *et al.*, 2017^34^	Academic teaching hospital, Groote Schuur Hospital, in the Western Cape	Observational	General medical patients who had been prescribed antibiotics (public)	To report, over 4 years: (i) antibiotic consumption; and (ii) cost of a public hospital ASP in SA	A comprehensive ASP consisting of online education, an antibiotic prescription chart and weekly antibiotic stewardship ward rounds	Total antibiotic consumption fell from 1046 DDDs per 1000 patient days in 2011 (control period) to 868 DDDs per 1000 inpatient days by 2013 and remained at similar levels for the next 2 years following introduction of the ASP Reductions in IV antibiotic use were noted, particularly for ceftriaxone Cost savings on antibiotics over 4 years adjusted for inflation totalled ZAR 3.2 million Laboratory tests and costs increased (total increased cost = ZAR 0.4 million) There was no difference in inpatient mortality or 30 day readmission rates following the interventions
Brink *et al.*, 2016^32^	47 private hospitals operated by the hospital group Netcare Ltd. in seven of the nine South African provinces	Observational	Netcare hospital patients (private)	Assess the implementation of an ASP in a setting with limited infectious disease resources	A pharmacist-driven, prospective audit and feedback strategy for AMS	116 662 patients receiving antibiotics at 47 hospitals during 104 weeks of standardized measurement and feedback were reviewed 7934 pharmacist interventions were recorded An estimated 1 in 15 prescriptions required intervention 3116 (39%) of 7934 pharmacist interventions dealt with excessive antibiotic duration ASP led to a decrease in average antibiotic DDDs per 100 patient days from 101.38 (95% CI 93.05–109.72) in the pre-intervention phase to 83.04 (74.87–91.22) in the post-intervention phase (*P* < 0.0001)
Brink *et al.*, 2017^25^	34 private hospitals operated by the hospital group Netcare Ltd. in seven of the nine South African provinces	Observational	Netcare hospital patients (private)	To implement an improvement model for PAP	A pharmacist-driven, prospective audit and feedback strategy involving change management and improvement principles	70 weeks of standardized measurements and feedback was conducted 24 206 surgical cases were reviewed Significant improvement (*P* < 0.0001) in compliance with all process measures from 66.8% (95% CI 64.8–68.7) to 83.3% (95% CI 80.8–85.8)
Bronkhorst *et al*., 2014^36^	Steve Biko Academic Hospital, Gauteng	Quantitative, cross-sectional, operational, prospective audit	All patients admitted to surgical and trauma ICU wards (public)	To describe the contributions of a clinical pharmacist in a surgical and trauma ICU as evidence for the necessity of an appointment of a full-time clinical pharmacist in the ICU	Implementation of AMS and drafting and implementation of antimicrobial guidelines in the ICU through the introduction of clinical pharmacy (assessment of prescribing patterns, drug-related interventions and time needed to provide clinical pharmaceutical care)	Of the total 181 interventions suggested, 70% were accepted and implemented 15.5% of interventions dealt with untreated medical conditions and, where indicated, 13.8% on inappropriate therapy or course and 11% on inappropriate doses or dosing frequency 28% of the 250 h that the pharmacist spent on the ward was dedicated to pharmaceutical care while 21% was directed toward ward rounds
Chunnilall *et al*., 2015^22^	Private anonymized hospital, KZN	Quantitative, retrospective analysis	Adult ICU patients (private)	To evaluate the prescribing patterns and adherence to STGs, EML, SAMF and IDSA guidelines	Surveillance of prescribing patterns and adherence to STGs, EML, SAMF and IDSA guidelines	28.8% of patients (*n* = 226) received antibiotics in ICU 58.5% (*n* = 131) required antibiotics Of these, 70.2% were prescribed treatment consistent with the guidelines Doses were correct in 91.1% of the sample Evidence of microbiological investigations in 61.2% of patients De-escalation was noted in only 13.1% of the 70.8% of cases 41.1% of patients received an antibiotic prescription without indication There was a lack of microbiological verification in 38.8% of patients, inaccurate drug choice in 29.8% of the subset for whom antibiotics were indicated and incorrect dosing in 8.9% of the subset for whom antibiotics were indicated
du Toit, 2015^38^	Critical care unit of an acute-care private hospital, Stellenbosch, Western Cape	Pre-and post-interventional	Critical care unit patients (private)	To identify the role of the critical care nurse in the implementation of an ASP	None	Nurses can play an important role in the implementation of an ASP and are a cost-efficient resource Nurses should be an essential part of an AMS team Additional team training regarding AMS and IPC is necessary
Gasson *et al*., 2018^27^	PHC facilities, Cape Town, Western Cape	Retrospective review of medical charts	Patients attending PHC facilities (public)	To evaluate: (i) prescribing in PHC facilities in the Cape Town Metro District; (ii) adherence to current national guidelines; and (iii) reasons why prescriptions were not adherent to guidelines	None	654 cases were included An antibiotic was prescribed in 68.7% Overall guideline adherence was 45.1% There was a significant difference in adherence between facilities Healthcare professional type and patient gender had no significant effect on adherence The main reasons for non-adherence to guidelines were an undocumented diagnosis (30.5%), antibiotic not required (21.6%), incorrect dose (12.9%), incorrect drug (11.5%) and incorrect duration of therapy (9.5%)
Hoffman *et al*., 2017^31^	Red Cross War Memorial Children’s Hospital, Cape Town, Western Cape	Phase 1: retrospective folder review; Phase 2: post analysis of a multicomponent education intervention	Convenience sample of the first 107 available patient folders; study population included both inpatients and outpatients less than 18 years old (public)	To conduct an MUE as a quality-improvement project on an item that met the following criteria: high expenditure, high usage, high risk or problem prone (inappropriate use)	MUE of nystatin oral drop usage; posters were designed to inform prescribers about the differential diagnoses of OC and contained an algorithm to follow when dealing with OC, based on the PHC level 2014 STG; in-service training sessions were held at various meetings to share the results of the MUE and to distribute the posters	Of the files reviewed, only 24.3% indicated oral thrush as the diagnosis 76% of nystatin prescriptions were for a condition that was not in the STGs 54% of prescriptions were for use during antibiotic therapy, 23% as prophylaxis after liver and renal transplant and 5% following caustic injury of the oesophagus
Mabila *et al*., 2016^23^	VVMH in Tzaneen, Limpopo Province	Retrospective cross-sectional census study	Paediatric patients (public)	To determine antibiotic prescribing patterns amongst healthcare professionals in a paediatric ward at VVMH	None	Ampicillin (64.7%) was the most commonly prescribed antibiotic, followed by gentamicin (47.4%) The most common conditions for which the antibiotics were prescribed were bronchopneumonia, diarrhoea and dehydration 69.1% of prescriptions were in accordance with STGs 66.1% of antibiotics were prescribed empirically and did not depend on culture, i.e. in most cases no laboratory results were requested
Matsitse *et al*., 2017^24^	Two correctional centres in the North West Province	Investigational descriptive study, including retrospective and prospective data	One male-only facility housing approximately 1500 inmates as well as awaiting-trial detainees; the second centre housed both male and female inmates (1400); both were PHC facilities (public)	To assess: (i) compliance with the 2008 PHC STGs/EML in the management of sexually transmitted infections; and (ii) potential factors contributing to the compliance and non-compliance with STGs	None	MUS, LAP and GUS were the three most common STIs Doxycycline (95.0%), ciprofloxacin (90.6%) and metronidazole (90.1%) were prescribed for most of the STIs Overall compliance with the 2008 PHC STGs/EML was 75.9% for MUS, 11.4% for LAP and 14.8% for GUS
Messina *et al*., 2018^26^	Four South African private hospitals (Johannesburg and Pretoria), Gauteng	Retrospective chart review	Adult patients aged >18 years who were prescribed IV colistin for at least 72 h (private)	To evaluate: (i) the current utilization of colistin in four private-sector SA hospitals; and (ii) opportunities to improve the appropriate use of colistin in the future	None	There was 99.0% compliance with obtaining a culture prior to antibiotic therapy, 93.5% compliance with prescription of a loading dose and 98.5% compliance regarding prescription of colistin in combination with another agent; overall composite compliance with the six colistin stewardship process measures was 82.0% Non-compliance related to inappropriate loading and maintenance doses, lack of adjustment according to renal function and lack of de-escalation following culture and susceptibility Significantly shorter durations of treatment were noted in patients who received higher loading doses (*P* = 0.040) and in those who received maintenance doses of 4.5 MU twice daily versus 3 MU three times daily (*P* = 0.0027) Compared with patients who survived, more patients who died received the 3 MU three times daily maintenance dose (*P* = 0.0037; phi coefficient 0.26)
Messina *et al.*, 2015^33^	33 private-sector Netcare hospitals in SA	Prospective multicentre study	Netcare hospital patients (private)	To measure the change in compliance with IV antimicrobial administration within 1 h following implementation of a pharmacist-driven hang-time process-improvement protocol	Pharmacist-led interventions followed by hang-time compliance assessment consisting of five stages	32 985 patients who received new IV antibiotic orders were assessed for hang-time compliance with first doses over a 60 week period 21 069 patients appropriately received first dose IV antibiotics within 60 min after prescription order, i.e. were hang-time compliant Hang-time compliance improved following the pharmacist-led intervention from 41.2% pre-intervention Week 1 (164/398) to 78.4% post-intervention Week 60 (480/612; P < 0.0001) Pharmacists’ patient review time improved during the final 4 weeks (1680) compared to the first 4 weeks (834; *P *< 0.0001)
Mthethwa and Matjila, 2018^30^	Medunsa Oral Health Centre, Gauteng	Retrospective, cross-sectional descriptive study of medical records	A portion of medical records of patients who received medication from the Medunsa Oral Health Centre (public)	To evaluate the antibiotic prescribing practices of dentists; surveillance of amount and class used and condition for which the antibiotic was prescribed	None	Antibiotics were prescribed for 65.5 % of the participants 59.1% of the prescriptions were for prophylactic use of antibiotics Of these, 65.5% were healthy patients with no history of a systematic illness 10.9% were HIV infected Routine extractions accounted for 54% of the perioperative prophylactic recipients 5.4% of the antibiotics prescribed were in the absence of a diagnosis Amoxicillin 500 mg three times daily was the most frequently prescribed antibiotic
Paruk *et al*., 2012^21^	South African private and public hospitals	Three-part prospective, descriptive study that included a 1 day point prevalence study to provide a ‘snapshot’ of events in the ICU	Study population comprised public- and private-sector hospitals in SA that were included in part 1 of the National Critical Care Audit; to ensure a true South African representation, all adult and paediatric ICUs in the private and public (tertiary, regional and community level) sectors were included (private and public)	To: (i) document antibiotic prescription practices in public and private ICUs in SA; and (ii) determine their relationship to patient outcomes		Therapeutic antibiotics were initiated in 182 (73.5%) of 248 recruited patients 54.9% received an inappropriate antibiotic initially De-escalation was practiced in 33.3% and 19.7% of the public and private sector patients, respectively Antibiotic duration was inappropriate in most cases An appropriate choice of antibiotic was associated with an 11% mortality, while an inappropriate choice was associated with a 27% mortality (*P *= 0.01) The mortality associated with appropriate or inappropriate duration of antibiotics was 17.6% and 20.6%, respectively (*P *= 0.42)
Ramsamy *et al.*, 2013^29^	TICU at the IALCH, a tertiary/quaternary public service institution in Durban, KZN	Prospective descriptive database review	All patients admitted to the TICU, IALCH who underwent mechanical ventilation during the study period (public)	To determine: (i) the spectrum of nosocomial pathogens in a level 1 TICU based on monthly surveillance and how frequently the initial empirical choice of antimicrobials was correct; and (ii) whether ultrabroad-spectrum combination therapy (U-bSCT) was warranted and, when used, how frequently it was actually necessary	Not applicable	Of 227 patients, 106 (46.6%) had 136 culture-positive isolates These included a total of 323 pathogens (201 Gram-negative, 119 Gram-positive, 3 *Candida albicans*) There were 19 species of Gram-negative pathogens, of which 56% comprised Enterobacteriaceae ESBL production was found in 6 of 31 (19%) *Escherichia coli* and 6 of 24 (25%) *Klebsiella* isolates Staphylococcal species accounted for 60% of the Gram-positive isolates, of which 18 were MRSA All *Candida* isolates were susceptible to fluconazole Despite positive cultures, antimicrobials were not prescribed for 21 patients who had no evidence of sepsis Excluding MDR *Acinetobacter* isolates, there were 87 (93.5%) appropriate and 6 (6.5%) incorrect prescriptions U-bSCT was employed for 11 patients but was necessary in only 2
Rout *et al*., 2017^37^	200 bed private hospital in KZN	Qualitative research approach; semi-structured interviews	Intensive/high care unit patients (private)	To understand the perceptions of AMS team members regarding the role of the ICU nurse in the AMS team	None	Participants from the different disciplines of the AMS team felt that the ICU nurse played an important role within the AMS team; four functions were identified as important and included organizational, advocacy, clinical and collaborative roles
van der Sandt *et al*., 2019^28^	1650 bed public anonymized hospital;358 bed private anonymized hospital, SA	Retrospective chart reviews	Paediatric patients undergoing surgery (public and private)	To evaluate compliance with South African SAP guidelines for paediatric patients undergoing surgery in surgical subspecialties (ENT, colorectal, urology and maxillofacial)	Adherence to the SAP guidelines	224 charts were reviewed (112 from each of the private and public sectors) The majority (*P* = 1.000) of patients received antibiotics in line with the SAP (77.3% and 100.0%, respectively, from the public and private hospitals) 21.1% and 45.9% of patients, respectively, received antimicrobials without an indication Full compliance to all five of the criteria (correct choice of antibiotic, dose, timing of administration, redosing and duration of use) were not met by either the public or private hospitals The majority of criteria were met in the teaching hospital (three out of five conditions or 58%); there is a need for quality-improvement interventions, surveillance and implementation of SAP guidelines

VVMH, Van Velden Memorial Hospital; KZN, KwaZulu-Natal; IALCH, Inkosi Albert Luthuli Central Hospital; OC, oral candida; MUS, male urethritis syndrome; LAP, lower abdominal pain; GUS, genital ulcer syndrome; SAMF, South African Medicine Formulary; MU, million units; ENT, ear, nose and throat; TICU, trauma ICU.

## Results

A comprehensive systematic title search of the databases (PubMed and Scopus) using the selected keywords ‘antimicrobial antibiotic stewardship South Africa’ yielded 192 articles and 38 articles, respectively. One hundred and eighty-one additional records were identified through the UKZN library WorldCat iCatalogue (*n* = 111), the FIDSSA website (*n* = 25), policy documents (*n* = 2), *SAMJ* (*n* = 4) and reference lists (*n* = 39) (Figure 1). In total, 411 records were identified for screening. Two hundred and sixty-three records remained after removal of duplicates. Screening of titles and abstracts identified 146 records. One hundred and seventeen articles were excluded for the following reasons: studies conducted outside SA (*n* = 45), IPC (*n* = 17), surveillance with no AMS intervention (*n* = 47), AMS in animals/agriculture (*n* = 3), AMS interventions that were carried out in the community (*n* = 2) and articles not related to AMS (*n* = 3). The full-text screening of the articles excluded a further 120 articles owing to lack of AMS interventions and AMS interventions not specific to SA (*n* = 2). Excluded articles were either reporting on AMS usage with no active AMS intervention, policy documents, a commentary, a debate form of report or reviews. This left 24 articles that were assessed for methodological quality using the MMAT checklist. Six articles were excluded for reasons such as poor or absent methodology. These articles did not meet any of the criteria on the MMAT checklist. A total of 18 eligible articles were included for the final analysis.

There were 17 quantitative descriptive studies and one qualitative study. The quality of the study methodology was graded according to four quality indicators as indicated in the MMAT methodology:^19^

- Qualitative studies [(1) sources of data, (2) process for analysing data, (3) deliberation of how the findings relate to the context, i.e. research setting and (4) deliberations of how the findings might be related to the researcher’s influence]
- Quantitative descriptive [(1) sampling strategy, (2) relation of the sample to the population understudy, (3) appropriateness of method and (4) acceptable response rate].

The studies were scored according to how well they met the methodology quality indicators. Thirteen studies (72%) matched all four criteria and five studies (28%) matched only two of the quality indicators. Of these five studies, two did not adequately describe the sampling strategy. In three of the studies it was difficult to relate the sample size chosen to the population under study and/or these studies lacked the following criteria: appropriateness of study method or an acceptable response rate.

There were seven papers from the private sector, nine papers from the public sector and two papers representing both the public and private sectors.

### Types of AMS initiatives or strategies

The studies revealed that healthcare facilities across both the private and public sectors in SA were in various stages of AMS implementation. Following content analysis in line with the objectives and inclusion criteria, the data was broadly divided into four significant AMS strategies/interventions:

- AMS intervention: prescription audits and usage
- AMS intervention: education and its impact
- Other AMS interventions
- The role of different healthcare professionals in AMS

Fifteen studies covered prescription audits and usage. Six studies incorporated education to foster AMS awareness. Five studies covered ASPs and 11 studies acknowledged the importance of the roles that different healthcare professionals play in a successful ASP.

#### AMS intervention: prescription audits and usage

Fifteen of the studies that were reviewed and selected audited antibiotic use.^21–35^ This included antibiotic prescribing patterns, frequency, the most commonly prescribed antibiotics and the indication for choice of antibiotic. Dosage, duration, route of administration, hang times and drug–bug match were also reviewed in various studies.^21–28^^,^^32–36^ Some studies also reported on adherence to South African treatment guidelines [national standard treatment guidelines (STGs)], the Essential Medicines List (EML),^22–24^^,^^27^ surgical antimicrobial prophylaxis (SAP)^28^ and rational prescribing.^22–24^^,^^27^ In addition to the local guidelines, one study further evaluated adherence to international guidelines (IDSA).^22^ Some of the studies also reported on the methodology used to confirm patient diagnosis, microorganism identification and antibiotic susceptibility testing, de-escalation and intravenous (IV)-to-oral switch in antimicrobial therapy.^21–23^ It was noted that studies were either carried out on selected wards or outpatient departments within a designated facility,^22^^,^^23^ or in more than one facility within the same private hospital chain.^25^^,^^26^^,^^32^

Two studies focused on the use of a specific antibiotic. The first identified a need for a specific colistin bundle^26^ and the other evaluated the usage of nystatin, which is a high-value, high-usage item.^31^ One study was carried out in a particular type of ward, i.e. ICUs across SA, which included the private and the public healthcare sectors.^21^ This study highlighted that antibiotic de-escalation was only practised in 33.3% and 19.7% of the public and private sector patients, respectively, and antibiotic duration was inappropriate in most cases.^21^ Gasson *et al.*^27^ looked at eight PHC facilities within the Cape Town Metro district. This study gave much broader insight into the prescribing tendencies within a specific vicinity. In general, adherence to treatment guidelines was only 45.1%. In 30.5% of cases the diagnosis was missing, 21.6% did not require an antibiotic, 12.9% had the incorrect dose, 11.5% the incorrect drug and 9.5% an incorrect duration of therapy. A number of studies showed that antibiotic prescribing was empirical rather than dependent on microbiological results.^22^^,^^23^ All these studies demonstrated that there were inappropriate antimicrobial prescribing practices (incorrect dose, duration, drug, lack of de-escalation, empirical prescribing and poor adherence to treatment guidelines) and a need for AMS.

#### AMS intervention: education and its impact

Education appeared to play a major role and was essential to the success of any ASP. In six studies, education took the form of either structured courses or more *ad hoc* educational ventures that were centred on specific AMS initiatives.^25^^,^^31–35^

In addition to antibiotic audits, some studies had an additional component of AMS education.^31^^,^^34^^,^^35^ These included educating prescribers on AMS and re-enforcement of usage of the STGs and EML. Combining educational interventions with antibiotic usage audits improved compliance in most institutions.^31^^,^^34^^,^^35^ As part of a quality-improvement project to evaluate the success of a medicines use evaluation (MUE), one study looked at a single high-expenditure item such as nystatin. An MUE before and after an educational initiative showed improved usage.^31^

#### Other AMS interventions

In conjunction with audits analysing antimicrobial use, some institutions are in the process of implementing or have implemented successful ASPs.^32^^,^^34^^,^^35^ Commendable examples (five studies) have been found in both the public^34^^,^^35^ and private^25^^,^^32^^,^^33^ sectors despite a resource-limited setting.

In the public sector, Boyles *et al.*^35^ introduced a dedicated antimicrobial prescription chart and instituted weekly antibiotic stewardship ward rounds in two medical wards in Groote Schuur Hospital. The volume and cost of antibiotics over this period were retrieved from electronic pharmacy records. The marker to determine antibiotic usage was DDDs. The outcomes of the intervention were assessed against baseline data obtained in the previous year. The outcome was a sizeable reduction in antibiotic volume (19.6%) and pharmacy budget (35%).^35^ The educational tool used was web-based, containing material regarding spectra of antibiotic coverage and an interactive case-based tutorial. The antibiotic prescription chart was designed to record three separate infection episodes. The episodes were coded as prophylactic (P), empirical (E) or definitive (D); the latter was based on microbiological culture and susceptibility. This prescription chart was used only for antibiotics and did not include antifungal or antiviral agents. Regular multidisciplinary antibiotic ward rounds were conducted by the core AMS team (infectious disease specialist, clinical microbiologist, IPC nurse and ward pharmacist) that was joined by a ward nurse and medical registrars responsible for those patients. Each patient was reviewed, the case discussed, an action plan agreed upon and education around stewardship conducted. Point prevalence audits on AMS prescription charts were performed once weekly for 4 weeks where use of charts as well as completion of all fields on the chart were audited. The institution of ward rounds and a dedicated AMS prescription chart reduced antibiotic use and costs without affecting mortality.^35^ Interestingly, a continuous assessment over 4 years from 2013 to 2016 showed that stewardship maintained a sustained reduction in antibiotic consumption and a cost saving of ZAR 3.2 million (approximately $0.21 million) was also noted.^34^

In the private sector, studies were carried out across multiple sites within a hospital network. The three pharmacist-led studies/initiatives were carried out at Netcare hospitals (a private hospital group in SA).^25^^,^^32^^,^^33^ Messina *et al.*^33^ carried out a prospective multicentre study across 33 hospitals to evaluate hang-time compliance of the initiation of first doses following new antibiotic orders. A guide was created to implement the different stages of the project. A hang-time educational poster was designed, followed by a 4 week implementation period that covered four stages: stage 1 looked at the initial hang-time compliance for a chosen ward (baseline); stage 2 consisted of pharmacist-led group education and training sessions, which included nurses; stage 3 included pharmacist-led monitoring and data collection of hang-time compliance using a standard template; and stage 4 involved submission of results to a study coordinator who tracked and collated results in real time with feedback. This initiative led to a reduction in hang times with 78.4% of patients receiving the first antibiotic dose within 1 h.^33^

Another pharmacist-led study aimed to assess/measure adherence to peri-operative antibiotic prophylaxis (PAP) guidelines. This was a prospective audit with feedback for the development of an improved model for PAP. During the pre-implementation phase, the PAP guideline and process measures were tested and refined at pilot sites, then rolled out during the post-implementation phase to 34 Netcare hospitals. The four process measures to determine adherence were: (i) antibiotic choice; (ii) dose; (iii) administration time; and (iv) duration. The study also included an educational component, which was carried out via institutional workshops. This initiative showed improved compliance with adherence to all four process measures.^25^

In 2016, Brink *et al.*^32^ looked at implementation of a pharmacist-driven ASP. This project was run across 47 Netcare hospitals. The pre-implementation phase observed baseline AMS interventions, followed by a stepwise implementation process at each hospital, which included interventions to reduce antibiotic consumption. The process measures used were prolonged duration, multiple antibiotics and redundant antibiotic cover. Once the model was established, the post-implementation phase assessed antibiotic consumption via DDDs and the change in antibiotic consumption at each hospital. Interestingly, 1 in 15 prescriptions required a pharmacist intervention. There was also a reduction in mean antibiotic DDDs from 101.38 (95% CI 93.05–109.72) to 83.04 (95% CI 74.87–91.22, *P* < 0.0001).^32^

#### The role of different healthcare professionals in AMS

Eleven studies explored the different roles of healthcare professionals in AMS.^25^^,^^29^^,^^30^^,^^32–38^

Although nurses play a prominent role in infection control, their role in AMS is not as clear. In a qualitative study conducted by Rout and Brysiewicz,^37^ the perceptions of nurses and other members of the healthcare team (clinicians, microbiologists and pharmacists) regarding the nurse’s role in AMS were explored. Four categories arose relating to how a nurse could be involved in AMS, which included the following:

- organizational (monitoring antimicrobial use, ‘Best Care…*Always!*’ bundle compliance, documentation of antibiotic treatment, follow-up on laboratory results and change in doctors’ instructions)
- advocacy (alerting clinicians to antibiotic duration to promote de-escalation)
- clinical (monitoring of patients to identify early signs of an infection and practicing IPC to prevent cross-infection)
- collaborative roles (with other nurses and healthcare professionals)^37^

du Toit^38^ explored the role of the critical care nurse in the implementation of AMS. In a resource-restrained environment, expanded roles of other healthcare professionals becomes increasingly important. The study demonstrated that nurses played an important role in AMS and IPC. Nurses are also a cost-effective resource that can be utilized.^38^

Much has been written about the clinical role of the pharmacist in AMS and their inclusion as part of the team in ward rounds.^25^^,^^32–36^ These studies show that interventions implemented by the pharmacist did improve patient care and pharmacists were instrumental in resolving medicine-related problems.^25^^,^^32^^,^^36^ Pharmacists play a vital role in monitoring the choice of antibiotic, duration, indication for use, reconciliation with biomarkers and patient clinical picture and diagnosis.^25^^,^^32^ Pharmacists also had a key role in analysis of the consumption and total costs of the antibiotics used.^25^^,^^32^^,^^34^

Microbiologists have a crucial role to play in AMS.^29^ Input offered by a clinical microbiologist is critical in the management of patients with infections. In daily practice, the clinical microbiologist has a diagnostic, consultative and advisory role, which extends into AMS activities. Following microbiological confirmation of infection, the clinical microbiologist is able to advise on appropriate antimicrobial therapy based on antimicrobial susceptibility results. Additionally, they are also able to make informed decisions about empirical antimicrobial therapy based on pathogen surveillance and AMR trends/patterns at a given institution over a period of time.^29^^,^^39^^,^^40^ This role is crucial; however, a number of investigators expressed concern that antibiotic treatment was not always informed by microbiology.^21–23^^,^^26^^,^^36^ In the studies conducted by Boyles *et al.*^34^^,^^35^ at Groote Schuur Hospital, consultant microbiologists were an integral part of the stewardship team participating on ward rounds. Brink *et al.*^32^ demonstrated that cultures obtained prior to the initiation of empirical therapy aided stewardship. Microbiology is a vital tool in preserving last-resort Gram-negative antibiotics.^26^^,^^29^ Pathogen surveillance is essential to confirming empirical antibiotic therapy and de-escalation.^29^ The use of regular microbiological surveillance can be an important tool in AMS to prevent indiscriminate use of broad-spectrum antimicrobials.^29^ Input offered by a clinical microbiologist is therefore critical in the management of patients with infections.

Stewardship principles should be practised by all healthcare professionals. A section of the healthcare sector that is largely overlooked is the dental sector. One study highlighted inappropriate prescribing and the need for more practical guidelines to aid dental prescribing.^30^

## Discussion

This scoping review revealed that facilities were in different stages of AMS implementation across both healthcare sectors in SA. Four significant AMS strategies/interventions emerged. These categories were: (i) AMS intervention: prescription audits and usage; (ii) AMS intervention: education and its impact; (iii) other AMS interventions; and (iv) the role of different healthcare professionals in AMS.

AMR is a fast-growing problem in low- and middle-income countries (LMICs). Data suggest that the rates of resistance are high but the extent of the problem is not fully understood owing to a paucity of data from certain regions.^2^^,^^11^ Together with overuse and misuse of antibiotics, LMICs have their own set of challenges including poverty, poor sanitation and inadequate IPC measures, in addition to low vaccination rates.^11^^,^^41^ Particular challenges to the implementation of AMS include lack of access to microbiological laboratories, poor availability of quality-assured antibiotics, lack of basic healthcare infrastructure and staff shortages.^11^

### Government policy and governance

As is the case globally, there is an increasing concern in SA over the rise in AMR. Coupled with this, SA has an appreciable burden of communicable and non-communicable diseases including drug-resistant bacteria, fungi, HIV and TB.^14^^,^^42–44^ SA is one of the members of the BRICS (Brazil, Russia, India, China and SA) nations. Collectively, BRICS is a very high consumer of antibiotics. In SA, there is also a lack of surveillance on antimicrobial consumption data at provincial, local, district and institutional levels. This is because there is a lack of integrated information systems connecting pharmacy, laboratory and clinical data.^44^ The Global Antibiotic Resistance Partnership—South Africa (GARP-SA) was formed in 2011 to obtain a situational analysis of AMR in SA. The South African Antibiotic Stewardship Programme (SAASP) was formed under FIDSSA. The WHO launched a Global Action Plan (GAP) on AMR in 2015.^45^ In response to insistence by SAASP and encouragement from the WHO for member states to develop a national plan to combat AMR,^43^ a national strategy framework for AMR was drafted by the South African NDOH in 2015.^5^^,^^8^ The three pillars of AMR governance are surveillance, IPC and AMS. The document provided a detailed guideline to instituting AMS at the facility level.^5^^,^^44^ A ministerial advisory committee (MAC), comprising key stakeholders was set up to oversee national surveillance, selection of antimicrobials in the EML, leadership and guidance for the implementation of AMS interventions at national, provincial, state and institutional level, advise on improvement strategies for IPC, vaccination programmes, and to provide guidance on core curricula for AMR and patient advocacy and awareness campaigns to promote appropriate use of antimicrobials in human and animal health.^6^^,^^44^ The organization of AMS interventions has begun to occur at provincial level. In the public sector, AMS interventions are mostly led from central hospitals with outreach and support at secondary and district hospitals.^7^ The South African EML and STGs were originally drawn up to promote appropriate prescribing.^46^ In addition to this, a pocket guide to clinical prescribing of antimicrobials was developed.^44^^,^^47^ These guides are routinely used in the public sector. The private sector, however, does not restrict or stipulate prescribing to specified guidelines.^7^

### Human resource challenges

The provision of high-quality healthcare is largely dependent on an adequate number as well as availability of suitably qualified healthcare professionals. An important challenge facing the delivery of healthcare in SA is the lack of sufficient human resources. These shortages are dire and exist across all professions: doctors, pharmacists and nurses. Coupled with this, there is a disproportionate spread of this workforce, with more healthcare professionals concentrating in the private sector in comparison with the public sector.^13^ Antibiotic stewardship teams are essential to tackling AMR and instituting AMS. In resource-abundant settings such as the USA and the UK these teams often comprise a multidisciplinary group including infectious diseases specialists, clinical microbiologists and clinical pharmacists, with all members suitably trained in stewardship principles.^48^ In LMICs this is not always the case.^48^ Despite the value of AMS in the reduction of AMR, ASPs in certain countries remain largely underfunded.^48^^,^^49^

In the face of these challenges, valiant efforts have been employed in both the private and public sectors to tackle the AMR issue in SA.

One of the easier interventions to implement is audits on antibiotic consumption. Often these studies form part of a baseline assessment of antimicrobial prescribing and usage. The data indicated that a number of facilities^21^^,^^22^^,^^24^^,^^26^^,^^27^^,^^50^^,^^51^ were in this stage where antibiotic usage and prescribing behaviour were audited. Prescribing behaviour was often assessed against adherence to SA’s STGs. The multisite studies^21^^,^^27^^,^^51^ were of particular value as they provided a snapshot of prescribing tendencies across a specific region. A study across eight PHC facilities demonstrated that adherence to treatment guidelines differed across the facilities and was poor, with less than 50% of the prescriptions adhering to treatment guidelines.^27^ The Prevalence of Infection in South African Intensive Care Units (PISA) study revealed that there were inappropriate prescribing practices across ICUs in both the public and private sectors.^21^ A study across six PHC facilities in the Johannesburg Metro district demonstrated that interventions, in this case AMS education, influenced the prescribing habits for the better.^51^ Audits of a particular antibiotic, e.g. colistin used for salvage therapy,^26^ and MUE on high-expenditure, high-usage items^31^ can provide value in improving stewardship around a particular antimicrobial agent.

A recently updated Cochrane review of AMS interventions established that educational interventions improved prescribing practices and specific competences in hospitals considerably.^12^ Around the world,^52–54^ including SA,^55^ evidence strongly suggests that medical students are ill-prepared for antimicrobial prescribing. The importance of educating medical students on the concept of AMR and the concepts of AMS is of paramount importance. Good prescribing practices amongst future healthcare practitioners is critical in combatting AMR. A recent study assessing the knowledge and attitudes of final-year SA medical students towards AMS^55^ found that only 33% felt confident in prescribing antibiotics whilst 95% felt they needed more education on the appropriate use of antibiotics.^55^ A similar outcome was obtained from a study on final-year pharmacy students. Although 83.5% of the respondents claimed that they knew what AMS was, 90% would like more education and training on AMS concepts.^56^ Education of all clinical professionals (pharmacists, nurses and clinicians) in AMS is equally important.^57–59^ Whilst educational programmes are available in the UK,^60^ the USA,^61^ Australia^62^ and many European countries,^63^ there is a lack of educational resources in LMICs.^52^ A massive open online course (MOOC) for AMS was an international initiative to address this need for support.^52^ A local initiative found that the use of a novel teaching method, a comic book depicting aspects of AMS, in the private sector improved pharmacy understanding of AMS principles.^64^ Two national stewardship training centres have been set up.^11^ In SA, courses and educational material are available on the FIDSSA website.^65^ It has, however, been identified that there is a lack of a coordinated standardized training programme for AMS in SA. Standardization of education within the undergraduate curriculum for all healthcare professionals could have a beneficial effect on reduction of AMR.^55^^,^^66^

A multidisciplinary AMS team is advocated by the SA NDOH.^6^^,^^8^ Much evidence for the success of an ASP depends on collaboration and a multidisciplinary team approach.^59^^,^^67^ The establishment of the role of each healthcare professional in AMS is vital to the programme’s success.^68^

IPC has clearly been the domain of nurses. A study exploring the role of nurses in AMS unearthed that nurses were well placed to take on additional roles beside IPC to support the prudent use of antimicrobials. These roles were organizational, advocacy, clinical and collaborative roles. Nurses have a significant support role in AMS interventions.^38^ Owing to the human resource challenge in SA, nurses have taken on additional roles such as prescribing. Task shifting has occurred with registered primary healthcare nurses in the public sector taking on prescribing roles with regard to antiretrovirals and antimicrobials.^69–71^ It therefore becomes crucial that nurses have sound AMS training.^66^ In a resource-limited setting like SA it is important to make use of the available resources. Pharmacists and registered nurses (RNs) are well positioned within the healthcare system to manage antimicrobial use for better patient outcomes.^72^

The clinical role of the pharmacist is important for the reduction of AMR in SA.^36^ Several studies show that interventions implemented by a non-specialist pharmacist did improve patient care and that pharmacists were instrumental in resolving medicine-related problems.^25^^,^^32^^,^^36^ Pharmacists play a major role in monitoring the choice of antibiotic, duration, indication for use, reconciliation with biomarkers, patient clinical picture and diagnosis.^25^^,^^32^^,^^36^ Pharmacists also play key roles in the auditing of antimicrobial usage and cost.^25^^,^^32^^,^^34^^,^^35^

The importance of microbiology support cannot be overstated. The role of microbiologists in the identification of resistant organisms and susceptibility testing is crucial to targeted prescribing. Microbiology interventions can also reduce the need for broad-spectrum prescribing and promote de-escalation.^29^^,^^73^ Surveillance plays an integral role in ASPs.^74^

A sector of the medical community that is largely overlooked when it comes to AMR is the dental sector. The data has shown that inappropriate prescribing does occur in the dental sector and that guidelines for more prudent dental prescribing is required.^30^^,^^75^

### AMS interventions

The initiation of a large-scale ASP can be daunting. The term ‘low-hanging fruit’ means the fruit that is found on the lower branches and is therefore more accessible. This terminology has applicability to ASPs where small quality-improvement projects are more achievable despite resource constraints.^76^ In the public sector, Boyles *et al.*^35^ found that the introduction of a dedicated prescription chart and multidisciplinary antibiotic ward rounds at Groote Schuur Hospital decreased antibiotic consumption and pharmacy costs. This was a sustainable intervention that was still successful 4 years on.^34^ At a rural hospital in George (Western Cape), a successful ASP was established owing to aid from a collaborative leadership initiative with the UK and outreach from Groote Schuur Hospital and the Western Cape Department of Health.^77^ For both Groote Schuur and George Hospitals, initial and ongoing AMS education played a major role in the success of the ASP. AMS was conducted by a multidisciplinary team and led by a physician. In Groote Schuur Hospital, AMS was led by an infectious diseases subspecialist.^34^ At George Hospital a dedicated young doctor/other healthcare professional was allocated to lead the programme for 6 month periods.^77^

In the private sector, most of the selected studies in this scoping review were from one hospital group (Netcare).^25^^,^^33^ These were pharmacist-led initiatives. A ‘hang-time’ initiative over a 60 week period across 33 hospitals showed that there was a substantial improvement in hang time compliance from 41.2% pre-intervention to 78.4% post-intervention.^33^ Another example of a pharmacist-driven initiative was a prospective audit and feedback for PAP adherence to guidelines. This was carried out across 34 Netcare hospitals over a period of 2.5 years. The study also included an educational component through institutional workshops. The intervention increased compliance with process measures (antibiotic choice, dose, administration time and duration) from 66.8% (95% CI 64.8–68.7) to 83.3% (95% CI 80.8–85.8).^25^ A recent study showed that ASPs via a three-phase (pre-intervention, intervention, post-intervention) process were successfully implemented across 47 South African Netcare hospitals. The importance of an ASP was seen as 1 in 15 prescriptions required intervention and there was a reduction in mean antibiotic DDDs per 100 patient days from 101.38 (95% CI 93.05–109.72) to 83.04 (74.87–91.22) in the post-implementation phase (*P* < 0.0001). These models also showed that pharmacist-led AMS interventions can thrive despite a resource-limited setting.^70^

### The public versus the private health sector

According to the literature, AMS initiatives are being carried out in both health sectors in SA. These range from small quality-improvement projects to fully set-up ASPs.

In the public health sector, initiatives are largely facility specific and usually physician led.^34^^,^^35^ There is evidence that outreach programmes are beneficial in helping to set up ASPs.^77^ In the public sector, the STG and EML documentation is also most likely used to guide prescribing,^78^ whereas in the private sector there is no restriction to specified guidelines.

Documents to guide governance, establishment and management of AMS at the hospital level is available to the public via the South African Department of Health website. These documents acknowledge that good governance, management and financial support is integral to a sustainable AMS.^8^^,^^79^^,^^80^ In the public sector, provincial AMS committees have been set up in the different provinces. This has been put in place to assist individual hospitals to set up their own AMS committees.^7^ To date, however, there is minimal information on AMS uptake and establishment at the facility level in the public sector. This knowledge is vital to inform government decision-making processes with regards to AMS establishment.

In the private sector, initiatives are carried out in an individual facility or across a hospital network. There appears to be more coordination and implementation of AMS interventions across hospital chains.^25^^,^^32^^,^^33^ Perhaps management support and promotion of prudent antimicrobial use ensures greater sustainability of AMS interventions in this sector.^81^^,^^82^ In the private sector, AMS is largely pharmacist led.^25^^,^^32^^,^^33^

Access to microbiology advice is available in both sectors. In the private sector, clinicians directly request pathology results from private laboratories. Clinical microbiologists are placed centrally within private laboratories and are able to access various private hospitals and private hospital groups.^83^^,^^84^ In the public sector, clinical microbiologists are stationed at certain provincial/state hospitals, with microbiological support also provided to the surrounding public hospitals. In this way most public-sector hospitals are either directly or indirectly in contact with a clinical microbiologist.^85^

Surveillance reports on MDR pathogens and their resistance patterns are compiled by clinical microbiologists in both the public and private sectors in SA.^39^^,^^40^^,^^86^ Surveillance information is shared between the private and public sectors. Data from both sectors are fed into the South African national surveillance system on AMR, a database managed by the National Institute for Communicable Diseases (NICD).^86^ These surveillance reports assist with informing empirical antimicrobial therapy for clinical conditions prior to microbiological confirmation.^40^^,^^87^

A gap exists in that there could be more knowledge-sharing between the two sectors as well as standardization of prescribing guidelines, which will go a long way to improve antimicrobial usage in SA.

### Limitations

This review only considered information published in the public domain. Studies prior to 2000 were not included. Some studies that might have brought operational change to a facility were excluded if methods were poor. Based on the pillars from the national AMS strategy, the authors only reviewed AMS and not surveillance and IPC. The authors also acknowledge that there may be AMS interventions occurring in both the public and private health sectors that are unaccounted for and there is a recommendation for more stakeholder engagement.

### Conclusions and recommendations

Amidst numerous challenges, AMS interventions can be successfully implemented, even if it is just the ‘low-hanging fruit’. Small interventions are having an impact on reducing AMR. The feasibility of an ASP, however, has to be contextualized regarding the challenges experienced within a specific healthcare setting.

Although the information derived from antibiotic usage audits is useful on its own, without enforcement of AMS principles these audits remain isolated and redundant. In those facilities where audits were combined with interventions, education and a continual assessment of initiatives, the ASPs thrived and were sustainable.

In a recent review on ASPs in LMICs, it has been acknowledged that specific guidelines should be set up to meet the needs of LMICs including SA.^11^ The following issues must be addressed:

- Easy access and availability of diagnostic tests
- Provision of education around AMR for healthcare workers at an undergraduate and in-service level
- Establishing and supporting (inter)national agencies for antimicrobial regulation
- Audits of antimicrobials usage
- Health systems strengthening
- Developing relationships between government, academia, professional bodies and civil society
- Formulating simple and sustainable AMS interventions for both hospital and community settings

The issue of resource constraints is a constant challenge in Africa. Therefore, innovative strategies are urgently needed to address AMS implementation needs at a nationwide level. Good governance and the standardization of healthcare professional training in AMS principles is vital to ASP sustainability. More should be done to foster links between academia and the different health sectors. International collaboration and support is vital to health systems strengthening and surveillance.

## Transparency declarations

Professor Essack is chairperson of the Global Respiratory Infection Partnership sponsored by an unrestricted educational grant from Reckitt and Benckiser Ltd., UK. All other authors: none to declare.

## Supplementary data

The Reviewer reports are available as Supplementary data at *JAC* Online.

## Supplementary Material

dlz060_Supplementary_Data
