# Supplementary material for: Antimicrobial stewardship in South Africa: a scoping review of the published literature
Source: JAC Antimicrob Resist. 2019 Nov 28;1(3):dlz060. doi: 10.1093/jacamr/dlz060 (PMC8210007; doi:10.1093/jacamr/dlz060)
Supplement: dlz060_Supplementary_Data [file dlz060_supplementary_data.zip › Reviewer report 1.docx]

**Reviewer report 1 (R0 – review of original submission)**

**PLEASE NOTE: JAC-AMR aims to publish your anonymous peer review comments with the final article for transparency. Please indicate in the box below whether you give your consent for us to publish your peer review comments for this article.**

Yes

**Synopsis**

**1. If the article contains original data, does the synopsis adequately reflect the manuscript as a whole, with the most important aspects described (of the introduction, methods and key results, and a balanced conclusion)? If NO, what is required to improve the synopsis? Please use the box below.**

Not applicable

**Introduction**

**2. Does the introduction provide the necessary background information for readers to understand the article? (e.g. does it describe the problem, current state of knowledge, etc.) If NO, what is required to improve it? Please use the box below**

No

**3. Is the research hypothesis and/or aims/objectives of the study clearly stated in the introduction? If NO, what is required to improve it? Please use the box below**

No

**4. Is there superfluous/unnecessary text in the introduction? (Only text that is required for readers to understand why the study has been done should be included). If YES, what text is superfluous and should be removed? Please use the box below**

No

Considering the answers above and any other comments/advice you may have, please indicate what is required to improve the Introduction:

It would be helpful to include an overview of uptake of AMS in hospitals in both sectors in SA if this is known.

There are 3 objectives but only the first one is examined in any detail with very little included on objectives 2 and 3. I suspect that this is because there is not much from the studies found to address these objectives. Suggest have a main objective and say if possible the following further objectives will be considered.

**Methods**

**5. Were the methods used suitably robust and appropriate for the research question(s) and/or the aims/objectives of the study? If NO, the article should be rejected, but please advise the authors accordingly in the free-text box below and complete the review in full**

No

**6. Are the methods described in enough detail so the reader can understand what was done and interpret the results and conclusions appropriately?**

No

**7. Are the methods described in language that a reader who does not specialize in the area of the study/methods used will understand? For example, are the statistical approaches used described in language that non-statistician healthcare professionals will understand? If NO, please indicate what is required to improve the language in the methods section in the box below**

Yes

Considering the above and any other comments/advice you may have, please indicate other aspects of the methods section that could be improved in the free-text box below)

It is usual in scoping reviews to use two databases but it appears that only Pubmed was used. Could the authors comment on the decision to only use one plus other sources.
For the initial screening of references found it appears that this was only done by one reviewer. Usual practice for scoping reviews is for two reviewers to be involved in this step of the process. Could the authors please clarify this and provide an explanation as to why only one reviewer was involved.

In the final stage of screening 6 studies were excluded as they did not meet the requirements of the quality assessment due to inappropriate methodology. It would be helpful to know what the issues were as of the final 18 some did not meet all quality indicators but had passed the quality assessment.

**Results**

**8. Given the methods used, are results described accurately, in enough detail and in an appropriate format to adequately inform the discussion section and the authors/readers conclusions? If NO, please indicate what is required to improve the results section in the box below**

No

**9. Based on what is described in the methods section, are data/results (that are likely to be available to the authors) missing from the current draft that you think should be presented to improve the manuscript?**

No

**10. Are Tables/Figures clear, required and do they add/complement the results text (they should not simply repeat what is in the text or vice versa)? If NO, please indicate what is required to improve the Tables/Figures in the box below**

Yes

Considering the above and any other comments/advice you may have, please indicate what is required to improve the results section:

Line 238 refers to de-escalation being 'practiced' could this be explained. Also refers to 'the study' but it is unclear which study they are referring to.
Line 241 - please describe what 'prescribing tendencies' were found.
Line 243-244 Please describe what 'inappropriate prescribing practices' were observed.
Line 250-256 Could details of what the education included be added.
Lines 284-286 Can info about role of microbiologists be linked back to the findings in the studies.
Line 287 Dental prescribing mentioned but I am unsure how this relates to hospital AMS.
Line 295 Could some details of what AMS programmes include be added.
Lines 299-320 Detailed information is provided on one small study in 2 wards. Seems disproportionate compared to details given on other studies.
Lines 321-331 and 332-339 What were the outcomes of these studies?

**Discussion**

**11. Are the discussion and any conclusion(s) reasonable given the stated hypothesis/aims/objectives and results? If NO, please indicate how to improve the discussion in the box below**

No

**12. Is there superfluous/unnecessary text in the discussion? (Only text that directly relates to the results and understanding thereof in the context of existing published literature and the limitations of the study should be included.) If YES, please indicate what should be removed to improve the discussion in the box below**

No

**13. Is the Limitations sub-section adequate and reflective of the actual limitations of the study? ? If NO, please indicate how to improve the Limitations section in the box below**

Yes

Considering the above and any other comments/advice you may have, please indicate how to improve the discussion section:

There is no mention of leadership for AMS and management support which are key factors in establshing AMS in hospitals.
More information about what microbiology support currently is and what the gaps are.

**General**

**14. Is referencing throughout the manuscript satisfactory? (for instance are there statements for which you believe a supporting reference should be cited?) If NO, please indicate how to improve referencing in the box below**

Yes

**15. Is the language used (i.e. spelling, grammar, etc.) of high enough quality to be published as is? If NO, please indicate how to improve the language within the article in the box below**

Yes

**16. Are ethics/other approvals appropriate/adequate and satisfactorily described? ? If NO, please indicate how to improve this in the box below**

Not applicable

Considering the above and any other comments/advice you may have, please advise the authors how to improve these aspects of the article.

**Opinion and review articles only**

**17. Please advise the authors in the box below on how to improve their opinion or review article:**

|  **Recommendation** | |
| --- | --- |
|  | Accept (as is with no revisions) |
|  | Minor revision (with anticipation of subsequent acceptance if revisions are adequate) |
|  | Major Revision |
|  | Reject |

| **Would you be willing to review a revision of this manuscript?** | |
| --- | --- |
|  | Yes |
|  | No |

**Comments**

 Comments to the Author

If you have any additional comments to the authors about any aspect of the manuscript not covered in the structured review above, please place these in the box below. If you have no additional comments, please say “No additional comments” in box.

No additional comments
