# Supplementary material for: Antimicrobial stewardship in South Africa: a scoping review of the published literature
Source: JAC Antimicrob Resist. 2019 Nov 28;1(3):dlz060. doi: 10.1093/jacamr/dlz060 (PMC8210007; doi:10.1093/jacamr/dlz060)
Supplement: dlz060_Supplementary_Data [file dlz060_supplementary_data.zip › Reviewer report 2.docx]

**Reviewer report 2 (R1 – review of revised submission)**

**PLEASE NOTE: JAC-AMR aims to publish your anonymous peer review comments with the final article for transparency. Please indicate in the box below whether you give your consent for us to publish your peer review comments for this article.**

Yes

**Synopsis**

**1. If the article contains original data, does the synopsis adequately reflect the manuscript as a whole, with the most important aspects described (of the introduction, methods and key results, and a balanced conclusion)? If NO, what is required to improve the synopsis? Please use the box below.**

Yes

It adequately reflect the manuscript as a whole, however a suggestion would be to add "in line with the AMR strategy of South Africa" to the end of the objectives to be aligned with what is described in the introduction and conclusions.

**Introduction**

**2. Does the introduction provide the necessary background information for readers to understand the article? (e.g. does it describe the problem, current state of knowledge, etc.) If NO, what is required to improve it? Please use the box below**

Yes

**3. Is the research hypothesis and/or aims/objectives of the study clearly stated in the introduction? If NO, what is required to improve it? Please use the box below**

Yes

**4. Is there superfluous/unnecessary text in the introduction? (Only text that is required for readers to understand why the study has been done should be included). If YES, what text is superfluous and should be removed? Please use the box below**

No

Considering the answers above and any other comments/advice you may have, please indicate what is required to improve the Introduction:

The reference from the WHO on antibiotics in the clinical pipeline states that there are very few antibiotics in the pipeline, but it does not say there are none. Thus this statement should be changed in the introduction to reflect this.

**Methods**

**5. Were the methods used suitably robust and appropriate for the research question(s) and/or the aims/objectives of the study? If NO, the article should be rejected, but please advise the authors accordingly in the free-text box below and complete the review in full**

Yes

**6. Are the methods described in enough detail so the reader can understand what was done and interpret the results and conclusions appropriately?**

Yes

**7. Are the methods described in language that a reader who does not specialize in the area of the study/methods used will understand? For example, are the statistical approaches used described in language that non-statistician healthcare professionals will understand? If NO, please indicate what is required to improve the language in the methods section in the box below**

Yes

Considering the above and any other comments/advice you may have, please indicate other aspects of the methods section that could be improved in the free-text box below)

Alignment of the terminology AMS interventions across the methodology section instead of AMS activities as currently both as used interchangeably.

The grouping of the types of AMS initiatives or strategies: I would suggest to label them along the following: 1) AMS intervention: prescription audits and usage, 2) AMS intervention: education and its impact, 3) Other AMS interventions, 4) the role of different health professionals in AMS. The reason for this adjustment is that both prescription audit and usage as well as education is a form of an AMS intervention.

**Results**

**8. Given the methods used, are results described accurately, in enough detail and in an appropriate format to adequately inform the discussion section and the authors/readers conclusions? If NO, please indicate what is required to improve the results section in the box below**

No

**9. Based on what is described in the methods section, are data/results (that are likely to be available to the authors) missing from the current draft that you think should be presented to improve the manuscript?**

No

**10. Are Tables/Figures clear, required and do they add/complement the results text (they should not simply repeat what is in the text or vice versa)? If NO, please indicate what is required to improve the Tables/Figures in the box below**

Yes

Considering the above and any other comments/advice you may have, please indicate what is required to improve the results section:

Paruk, 2013: would state PPS as the AMS intervention. It would be good to include an introductory paragraph for the results before the table and the more in depth description. Include the number for each type of intervention e.g. change vast majority of studies reviewed and selected audited antibiotic use, to the actual number of the studies. Same for the rest.

A current are to consider revising is the groupings, prescription audit and feedback is an AMS interventions as well as education. I would suggest to restructure as mentioned in the methodology comments or otherwise at a minimum provide sub titles for the AMS interventions sections and bring the audit and feedback under this section. One could distinguish between AMS interventions and AMS programmes.

**Discussion**

**11. Are the discussion and any conclusion(s) reasonable given the stated hypothesis/aims/objectives and results? If NO, please indicate how to improve the discussion in the box below**

Yes

**12. Is there superfluous/unnecessary text in the discussion? (Only text that directly relates to the results and understanding thereof in the context of existing published literature and the limitations of the study should be included.) If YES, please indicate what should be removed to improve the discussion in the box below**

No

**13. Is the Limitations sub-section adequate and reflective of the actual limitations of the study? ? If NO, please indicate how to improve the Limitations section in the box below**

Yes

Considering the above and any other comments/advice you may have, please indicate how to improve the discussion section:

**General**

**14. Is referencing throughout the manuscript satisfactory? (for instance are there statements for which you believe a supporting reference should be cited?) If NO, please indicate how to improve referencing in the box below**

Yes

**15. Is the language used (i.e. spelling, grammar, etc.) of high enough quality to be published as is? If NO, please indicate how to improve the language within the article in the box below**

Yes

**16. Are ethics/other approvals appropriate/adequate and satisfactorily described? ? If NO, please indicate how to improve this in the box below**

Yes

Considering the above and any other comments/advice you may have, please advise the authors how to improve these aspects of the article.

**Opinion and review articles only**

**17. Please advise the authors in the box below on how to improve their opinion or review article:**

|  **Recommendation** | |
| --- | --- |
|  | Accept (as is with no revisions) |
|  | Minor revision (with anticipation of subsequent acceptance if revisions are adequate) |
|  | Major Revision |
|  | Reject |

| **Would you be willing to review a revision of this manuscript?** | |
| --- | --- |
|  | Yes |
|  | No |

**Comments**

 Comments to the Author

If you have any additional comments to the authors about any aspect of the manuscript not covered in the structured review above, please place these in the box below. If you have no additional comments, please say “No additional comments” in box.

This is a very and important study, however it would be strengthened by putting numbers to the actual summary of interventions and their discussion. In addition by being more straight forward in the categorization a) AMS interventions, b) education, c) role of health-care providers this will strengthen the journal publication. In addition to add a paragraph to clearly identify the gaps.
